# Distinct mutations with different inheritance mode caused similar retinal dystrophies in one family: a demonstration of the importance of genetic annotations in complicated pedigrees

**DOI:** 10.1186/s12967-018-1522-7

**Published:** 2018-05-29

**Authors:** Xue Chen, Xunlun Sheng, Yani Liu, Zili Li, Xiantao Sun, Chao Jiang, Rui Qi, Shiqin Yuan, Xuhui Wang, Ge Zhou, Yanyan Zhen, Ping Xie, Qinghuai Liu, Biao Yan, Chen Zhao

**Affiliations:** 1 Department of Ophthalmology, State Key Laboratory of Reproductive Medicine, The First Affiliated Hospital of Nanjing Medical University, Nanjing, China; 2 Department of Ophthalmology and Vision Science, Eye & ENT Hospital, Shanghai Medical College, Fudan University, Shanghai, China; 3 Key Laboratory of Myopia of State Health Ministry (Fudan University) and Shanghai Key Laboratory of Visual Impairment and Restoration, Shanghai, China; 4 Department of Ophthalmology, Ningxia Eye Hospital, People Hospital of Ningxia Hui Autonomous Region (First Affiliated Hospital of Northwest University for Nationalities), Yinchuan, China; 5 Department of Ophthalmology, Children’s Hospital of Zhengzhou, Zhengzhou, China

**Keywords:** Retinitis pigmentosa, Genetic heterogeneity, Next generation sequencing, Mutation, *OFD1*, *C8ORF37*, *TULP1*, *RP1*, Consanguinity

## Abstract

**Background:**

Retinitis pigmentosa (RP) is the most common form of inherited retinal dystrophy presenting remarkable genetic heterogeneity. Genetic annotations would help with better clinical assessments and benefit gene therapy, and therefore should be recommended for RP patients. This report reveals the disease causing mutations in two RP pedigrees with confusing inheritance patterns using whole exome sequencing (WES).

**Methods:**

Twenty-five participants including eight patients from two families were recruited and received comprehensive ophthalmic evaluations. WES was applied for mutation identification. Bioinformatics annotations, intrafamilial co-segregation tests, and in silico analyses were subsequently conducted for mutation verification.

**Results:**

All patients were clinically diagnosed with RP. The first family included two siblings born to parents with consanguineous marriage; however, no potential pathogenic variant was found shared by both patients. Further analysis revealed that the female patient carried a recurrent homozygous *C8ORF37* p.W185*, while the male patient had hemizygous *OFD1* p.T120A. The second family was found to segregate mutations in two genes, *TULP1* and *RP1*. Two patients born to consanguineous marriage carried homozygous *TULP1* p.R419W, while a recurrent heterozygous *RP1* p.L762Yfs*17 was found in another four patients presenting an autosomal dominant inheritance pattern. Crystal structural analysis further indicated that the substitution from arginine to tryptophan at the highly conserved residue 419 of TULP1 could lead to the elimination of two hydrogen bonds between residue 419 and residues V488 and S534. All four genes, including *C8ORF37*, *OFD1*, *TULP1* and *RP1*, have been previously implicated in RP etiology.

**Conclusions:**

Our study demonstrates the coexistence of diverse inheritance modes and mutations affecting distinct disease causing genes in two RP families with consanguineous marriage. Our data provide novel insights into assessments of complicated pedigrees, reinforce the genetic complexity of RP, and highlight the need for extensive molecular evaluations in such challenging families with diverse inheritance modes and mutations.

**Electronic supplementary material:**

The online version of this article (10.1186/s12967-018-1522-7) contains supplementary material, which is available to authorized users.

## Background

Retinitis pigmentosa (RP, MIM: 268000), the most common form of inherited retinal degenerations, affects over one million individuals globally [1, 2]. Night blindness is usually the initial symptom for RP, followed by subsequent visual field constriction, and eventual vision loss. RP is featured by great clinical heterogeneities. Its onset age ranges from early childhood to mid-adulthood. Inter- and intra-familial phenotypic diversities caused by the same RP causing mutations have also been revealed [3–5]. Thus, clinical diagnose for RP patients are sometimes challenged by its wide phenotypic spectrum and under certain conditions, like in a young patient without fully onset RP phenotypes. In such situations, molecular testing could help to address the clinical ambiguity in RP diagnosis. RP also shows high genetic heterogeneity. To date, 83 RP causing genes involving hundreds of mutations have been identified (RetNet). Next-generation sequencing (NGS), enabling simultaneous parallel sequencing of numerous genes with high efficiency, is an efficient tool for molecular diagnosis of RP [2, 4]. Genetic annotations with NGS promote better clinical assessments and gene therapy, and therefore should be recommended for RP patients. However, pedigrees with puzzling inheritance patterns could sometimes confuse the genetic diagnoses. Herein, we described the genotypic and phenotypic findings in two complicated RP pedigrees using NGS. Distinct inheritance patterns and RP causing genes/mutations were found in both families.

## Methods

### Sample collection and clinical assessments

**Fig. 1:**
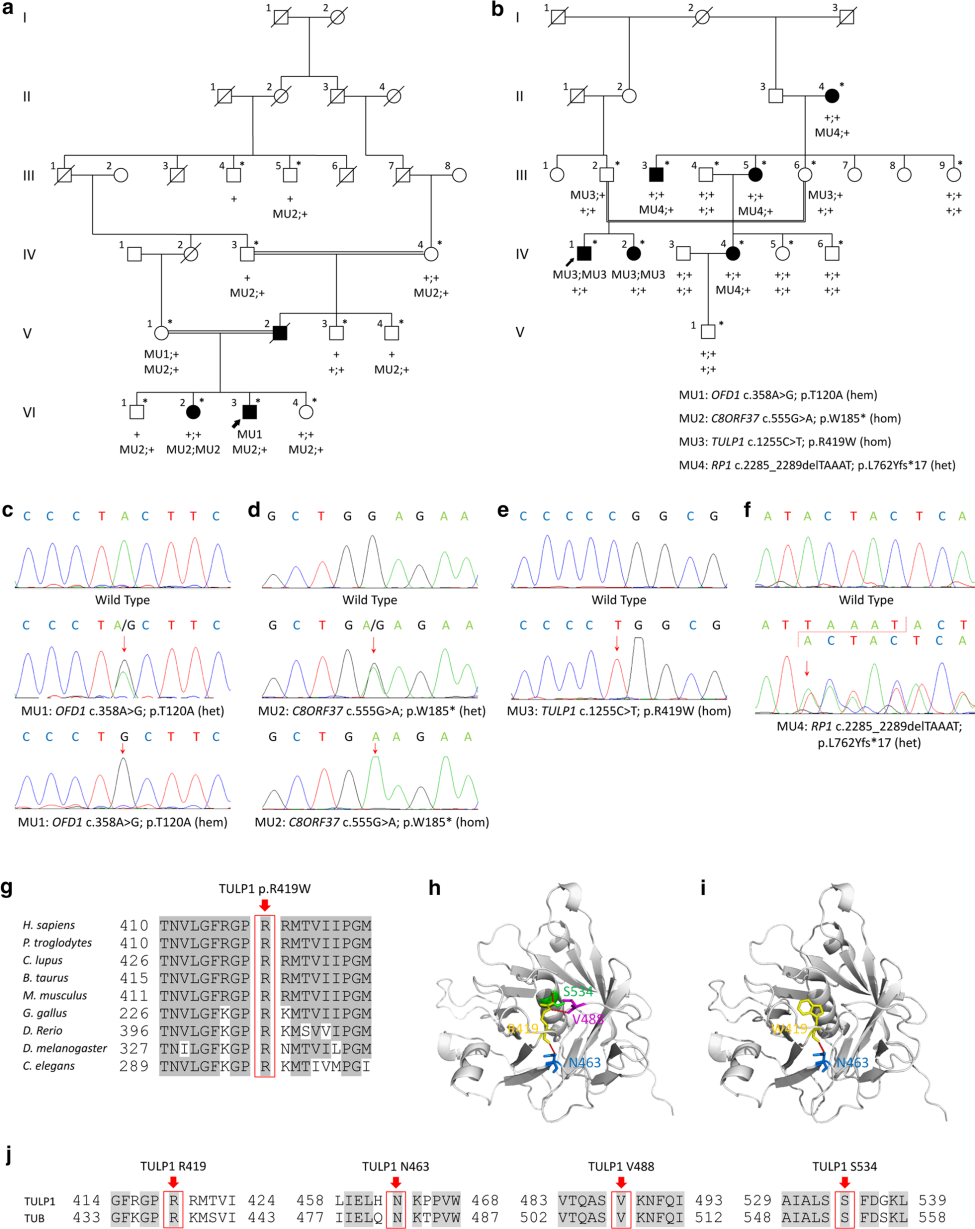
Family pedigrees and genetic annotations of identified mutations. **a** Pedigree of family A. Included participants are indicated by asterisk. **b** Pedigree of family B. Included participants are indicated by asterisk. **c**–**f** Sequence chromatograms of identified mutations, including *OFD1* c.358A>G (**c**), *C8ORF37* c.555G>A (**d**), *TULP1* c.1255C>T (**e**), and *RP1* c.2285_2289delTAAAT (**f**). **g** Orthologous protein sequence alignment of TULP1 from human (*H. sapiens*), chimpanzees (*P. troglodytes*), dogs (*C. lupus*), cows (*B. taurus*), rats (*M. musculus*), chickens (*G. gallus*), zebrafish (*D. rerio*), fruit flies (*D. melanogaster*), and worms (*C. elegans*). Conserved residues are shaded. The mutated residue 419 is boxed and indicated. **h**, **i** Crystal structural analysis of the wild type (**h**) and mutant (**i**) TULP1 protein. Hydrogen bonds between residue 419 and residues V488 and S534 were eliminated due to the substitution from arginine to tryptophan. **j** Conservational analysis of residues TULP1 R419, N463, V488 and S534 between TULP1 and TUB proteins

Our study, conformed to the Declaration of Helsinki, was approved and prospectively reviewed by the local ethics committee of People Hospital of Ningxia Hui Autonomous Region (No. 10 [2017]). Eleven participants from family A (Fig. 1a) and 14 participants from family B (Fig. 1b) were recruited from the People’s Hospital of Ningxia Hui Autonomous Region. Written informed contents were obtained from all participants or their legal guardians before their enrollments. Peripheral blood samples were collected from all 25 participants for genomic DNA extraction. Family history and consanguineous marriages were carefully reviewed. Medical records were obtained from all participants. Each participant received general ophthalmic evaluations, while comprehensive ophthalmic examinations were selectively conducted on the eight included patients. Another 150 Chinese healthy controls free of major ocular problems were recruited with their blood samples donated.

### NGS approach and bioinformatics analyses

To reveal the disease causing mutation in the two families, we selectively performed whole exome sequencing (WES) on three participants in family A (A-IV:3, A-VI:2 and A-VI:3) and two patients in family B (B-III:4 and B-IV:1). WES was conducted with the 44.1 megabases SeqCap EZ Human Exome Library v2.0 (Roche NimbleGen, Madison, WI) for enrichment of 23588 genes on patients from family A [6], and with SureSelect Human All Exon V6 60 Mb Kit (Agilent Technologies, Santa Clara, CA) on patients from family B [7]. Briefly, qualified genomic DNA samples were randomly sheared by Covaris into 200–250 base pair (bp) fragments. Fragments were then ligated with adapters to both ends, amplified by ligation-mediated polymerase chain reaction (LM-PCR), purified, and hybridized. Non-hybridized fragments were then washed out. Quantitative PCR was further applied to estimate the magnitude of enrichment of both non-captured and captured LM-PCR products. Each post-capture library was then loaded on an Illumina Hiseq 2000 platform for high-throughput sequencing.

Raw data were initially processed by CASAVA Software 1.7 (Illumina) for image analysis and base calling. Sequences were generated as 90 bp pair-end reads. Reads were aligned to human h19 genome using SOAPaligner (http://www.soap.genomics.org.cn) and Burrows-Wheeler Aligner (BWA; http://www.bio-bwa.sourceforge.net/). Only mapped reads were included for subsequent analysis. Coverage and depth were determined based on all mapped reads and the exome region. Atlas-SNP2 and Atlas-Indel2 were applied for variant calling [8]. Variant frequency data were obtained from the following six single nucleotide polymorphism databases, including dbSNP144 (http://www.hgdownload.cse.ucsc.edu/goldenPath/hg19/database/snp135.txt.gz.), HapMap Project (ftp://ftp.ncbi.nlm.nih.gov/hapmap), 1000 Genome Project (ftp://ftp.1000genomes.ebi.ac.uk/vol1/ftp), YH database (http://yh.genomics.org.cn/), Exome Variant Server (http://www.evs.gs.washington.edu/EVS/), and Exome Aggregation Consortium (http://exac.broadinstitute.org/). Variants with a minor allele frequency of over 1% in any of the above databases were discarded. Sanger sequencing was employed for mutation validation and prevalence test in 150 additional controls using a previously defined protocol [9]. Primer information is detailed in Additional file 1: Table S1 and Additional file 2: Table S2.

### In silico analysis

We applied vector NTI Advance™ 2011 software (Invitrogen, Carlsbad, CA) to analyze the conservation of the mutated reside by aligning protein sequence of human TULP1 (ENSP00000229771) with sequences of the following orthologues proteins: *P. troglodytes* (ENSPTRP00000030898), *C. lupus* (ENSCAFP00000001922), *B. taurus* (ENSBTAP00000055698), *M. musculus* (ENSMUSP00000049070), *G. gallus* (ENSGALP00000010281), *D. rerio* (ENSDARP00000099556), *D. melanogaster* (FBpp0088961), and *C. elegans* (F10B5.4). Crystal structural modeling of the wild type and mutant TULP1 proteins were constructed with SWISS-MODEL online server [10, 11], and displayed with PyMol software.

## Results

### Clinical findings

**Fig. 2:**
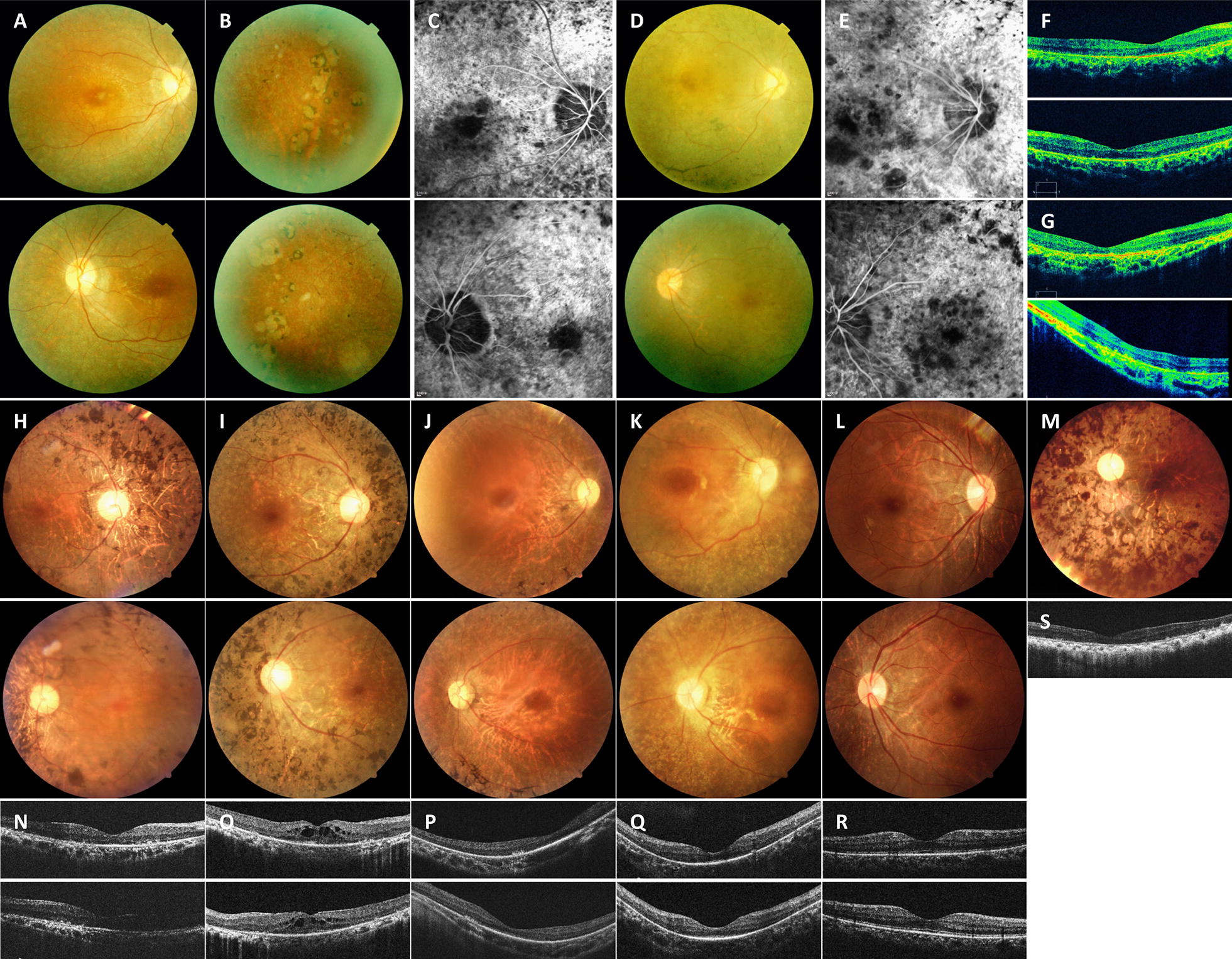
Ophthalmic presentations of included patients. **A**, **B** Fundus presentations of patient A-VI:3 (age 24, carrying *OFD1* c.358A>G) indicate waxy optic disc, attenuated retinal arterioles, macular degeneration, bone spicule-like pigments and atrophy of RPE and choroid in the peripheral retina. **C** Fundus fluorescein angiography (FFA) of patient A-VI:3 notices a combination of speckled hypofluorescent and hyperfluorescent changes in both macular and peripheral retina. **D** Fundus photos of patient A-VI:2 (age 27, carrying *C8ORF37* c.555G>A) show similar presentations to patient A-VI:3, but with more intensive pigmentations. **E** FFA of patient A-VI:2 also demonstrates intensive speckled changes of both hypofluorescence and hyperfluorescence. **F** OCT results of patient A-VI:3 indicate attenuated outer nuclear layer (ONL) and RPE with remarkable loss of inner segments (IS) and outer segments (OS). **G** OCT results of patient A-VI:2 show complete loss of IS and OS. **H** Patient B-III:3 (age 59, carrying *RP1* c.2285_2289delTAAAT) has a waxy optic disc, attenuated retinal arterioles, mild macular degeneration, and intensive bone spicule-like pigment deposits in the mid-peripheral retina of both eyes. **I** Patient B-III:5 (age 54, carrying *RP1* c.2285_2289delTAAAT) shows typical RP fundus similar to patient B-III:3, including intensive pigmentations and macular degeneration. **J** Fundus of patient B-IV:1 (age 27, carrying *TULP1* c.1255C>T) demonstrates attenuated retinal vessels, a waxy optic disc, remarkable macular degeneration, and diffuse pigment deposits in the periphery retina of both eyes. **K** Patient B-IV:2 (age 24, carrying *TULP1* c.1255C>T) shows similar fundus presentation to patient B-IV:1, presenting maculopathy and diffused pigmentations. **L** Slight waxy pallor of the optic disc and diffuse pigment deposits in the peripheral retina are revealed in the fundus of patient IV:4 (age 31, carrying *RP1* c.2285_2289delTAAAT). **M** Patient II:4 (age 80, carrying *RP1* c.2285_2289delTAAAT) shows typical RP fundus with intensive pigment deposits. **N** OCT results of patient B-III:3 indicate attenuated ONL and RPE with loss of IS and OS. **O** Thickened ONL with cystic cavities in the macular region were noticed by OCT in patient B-III:5. **P** OCT examinations of patient B-IV:1 demonstrate attenuated ONL and RPE with complete loss of IS and OS. **Q** Patient B-IV:2 shows similar OCT results to patient B-IV:1, including attenuated ONL and RPE, and loss of IS/OS. **R** Slightly attenuated ONL is presented in patient B-IV:4. **S** Typical RP presentations are revealed in patient B-II:4, demonstrating attenuated ONL and RPE with loss of IS and OS

Two patients from family A, A-VI:2 and A-VI:3, and six patients from family B, B-II:4, B-III:3, B-III:5, B-IV:1, B-IV:2 and B-IV:4, were included in the present study with their clinical details summarized in Table 1. Ophthalmic features of patient A-V:2 were obtained according to his medical records, and were presented in Table 1. All patients from the two families were clinically diagnosed with RP. In family A, all three patients had early onset nyctalopia and rapid disease progress. Best corrected visual acuity was light perception for both patients A-VI:2 and A-VI:3 at their last visit to our hospital at the ages of 25 and 24 respectively. Typical RP presentations and macular degeneration were detected upon their ophthalmic evaluations (Fig. 2A–G and Table 1). In family B, RP onset ages ranged from early childhood to 50 years old (Table 1). RP progression also varied among the 6 patients. Patients B-IV:1 and B-IV:2 reported to have nyctalopia since early childhood, while the other four patients showed RP symptoms elder than 30-year-old. On examination, typical RP presentations were detected for all 6 patients, while patient B-II:4 also had chronic angle closure glaucoma in her right eye (Fig. 2H–S). Noteworthy, all 6 patients presented mild to severe cataracts (Table 1). Patient B-III:3 received bilateral cataract surgeries 2 years ago. No systemic defect was noticed in any of the included patients.

**Table 1:** Clinical features of attainable patients

Family member ID	RP causative gene	Age (year)/sex	Onset age (year)	Night blindness	Cataract		BCVA (logMAR)		Fundus appearance								ERG	
									O.D.				O.S.					
					O.D.	O.S.	O.D.	O.S.	MD	OD	AA	PD	MD	OD	AA	PD	O.D.	O.S.
A-V:2^a^	–	–	10	Yes	–	–	LP	LP	–	–	–	–	–	–	–	–	–	–
A-VI:2	*C8ORF37*	25/F	8	Yes	No	No	LP	LP	Yes	Waxy	Yes	Yes	Yes	Waxy	Yes	Yes	D	D
A-VI:3	*OFD1*	24/M	2	Yes	No	No	LP	LP	Yes	Waxy	Yes	Yes	Yes	Waxy	Yes	Yes	D	D
B-II:4	*RP1*	80/F	50	Yes	Severe	Severe	NLP	LP	–	–	–	–	Yes	Waxy	Yes	Yes	–	D
B-III:3	*RP1*	59/M	30	Yes	IOL	IOL	0.6	0.25	Yes	Waxy	Yes	Yes	Yes	Waxy	Yes	Yes	D	D
B-III:5	*RP1*	54/F	35	Yes	Mild	Mild	0.3	0.3	Yes	Waxy	Yes	Yes	Yes	Waxy	Yes	Yes	D	D
B-IV:1	*TULP1*	27/M	EC	Yes	Moderate	Moderate	0.15	0.2	Yes	Waxy	Yes	Yes	Yes	Waxy	Yes	Yes	D	D
B-IV:2	*TULP1*	24/F	EC	Yes	Moderate	Moderate	0.3	0.3	Yes	Waxy	Yes	Yes	Yes	Waxy	Yes	Yes	D	D
B-IV:4	*RP1*	31/F	–	Yes	No	No	0.5	0.8	No	No	No	Yes	No	No	No	Yes	R	R

*F* female, *M* male, *EC* early childhood, *BCVA* best corrected visual acuity, *logMAR* logarithm of the minimum angle of resolution, *O.D.* right eye, *O.S.* left eye, *IOL* intraocular lens, *LP* light perception, *NLP* non-light perception, *MD* macular degeneration, *OD* optic disk, *AA* artery attenuation, *PD* pigment deposits, *ERG* electroretinography, *D* diminished, *R* reduced

^a^This patient is deceased. His clinical features are obtained based on his medical records

### Genetic assessments

To identify the pathogenic mutations, WES with high quality was selectively performed on individuals A-IV:3, A-VI:2, and A-VI:3 from family A (mean coverage: 98.16%; mean depth: 70.89×), and patients B-III:5 and B-IV:1 from family B (mean coverage: 98.32%; mean depth: 104.66×). NGS data were summarized in Additional file 3: Table S3. Exon-specific coverage report of all known RP genes was presented in Additional file 4: Table S4. For family A, patients A-VI:2 and A-VI:3 were born to parents with consanguineous marriage, supporting potential autosomal recessive inheritance. WES identified 10 homozygous variants and 6 compound heterozygous variants shared by patients A-VI:2 and A-VI:3 (Additional file 1: Table S1). However, Sanger sequencing revealed no variant co-segregated with the disease phenotype. We thus hypothesized that the two patients may have distinct RP causing mutations. Based on WES data, patient A-VI:2 carried a recurrent homozygous *C8ORF37* mutation c.555G>A (p.W185*; Fig. 1d and Table 2), while patient A-VI:3 had a novel hemizygous *OFD1* mutation c.358A>G (p.T120A; Fig. 1c and Table 2).

**Table 2:** Mutations identified in this study

Gene	Variation		Status	Bioinformatics analysis			Reported or Novel	Population prevalence (allele count)		
	Nucleotide	Amino acid		SIFT	PolyPhen	PROVEN		rs no.	gnomAD	EXAC
*C8ORF37*	c.555G>A	p.W185*	Hom	NA	NA	NA	Novel	rs748014296	2/246148	1/121412
*OFD1*	c.358A>G	p.T120A	Hem	0.63 (tolerated)	0.006 (benign)	− 0.616 (netural)	Novel	rs755625951	4/178544	1/121388
*TULP1*	c.1255C>T	p.R419W	Hom	0 (damaging)	1 (probably damaging)	− 7.976 (deleterious)	Novel	rs775334320	12/217192	6/121222
*RP1*	c.2285_2289delTAAAT	p.L762Yfs*17	Het	NA	NA	NA	Novel	NA	NA	NA

*Hom* homozygous, *Hem* hemizygous, *Het* heterozygous, *NA* not available

SIFT: http://sift.bii.a-star.edu.sg/; PolyPhen: http://genetics.bwh.harvard.edu/pph2/; PROVEN: http://provean.jcvi.org/index.php; gnomAD: http://gnomad.broadinstitute.org/; EXAC: http://exac.broadinstitute.org/

As to family B, WES revealed one homozygous variant and 18 compound heterozygous variants shared by patients B-III:4 and IV:2 (Additional file 2: Table S2), while no variant was validated co-segregated with the disease phenotype. According to the family pedigree, patients B-IV:1 and B-IV:2 were born to unaffected parents with consanguineous marriage, indicating a potential autosomal recessive inheritance pattern. However, the RP phenotypes of patients B-III:3 and B-III:4 were likely inherited from the affected mother B-II:4, suggesting a dominant inheritance mode. Upon this hypothesis, a novel homozygous *TULP1* mutation c.1255C>T (p.R419W; Fig. 1e and Table 2) was revealed as RP causative for patients B-IV:1 and B-IV:2, and a recurrent heterozygous *RP1* mutation c.2285_2289delTAAAT (p.L762Yfs*17; Fig. 1f; Table 2) was found in patients B-II:4, B-III:3 and B-III:4. The mutated residue R419 in TULP1 was highly conserved among all tested species (Fig. 1g). Crystal structures of the wild type and mutant TULP1 proteins were obtained based on human TUB protein (Protein Data Bank ID: 1S31) with a sequence identify of 75.19 and a sequence similarity of 0.54. Our data suggested that the substitution from arginine to tryptophan at residue 419 would lead to the elimination of two hydrogen bonds between residue 419 and residues V488 and S534 (Fig. 1h, i), further supporting that this mutation would disturb the tertiary structure of TULP1 and interrupt its function. Residues R419, N463, V488 and S534 were conserved between TULP1 and TUB proteins (Fig. 1j). All four mutations identified in the two families segregated with the disease phenotype (Fig. 1a, b), and were confirmed absent in 150 Chinese controls free of major ocular problems.

## Discussion

RP is a genetically heterogeneous disease with 83 disease causative genes and hundreds of mutations. In this report, molecular test reveals the coexistence of mutations affecting distinct RP causing genes in two RP families, thus providing novel insights into genetic assessments in complicated pedigrees. Among the four mutations identified in the two families, two were novel (*OFD1* p.T120A and *TULP1* p.R419W) and two were recurrent (*C8ORF37* p.W185* and *RP1* p.L762Yfs*17 [Human Gene Mutation Database ID: CD991855]).

*OFD1* mutations have been reported to cause X-linked recessive Joubert syndrome, orofaciodigital syndrome and isolated RP (Table 3) [12, 13]. OFD1, protein encoded by the *OFD1* gene, is a crucial component of the centrioles. OFD1 is involved in ciliogenesis regulation and exhibits neuroprotective roles [14]. Herein, a hemizygous *OFD1* missense mutation is associated with a severe form of RP presenting early onset age and fast disease progression. *C8ORF37* mutations correlate with a wide spectrum of autosomal recessive retinopathies ranging from RP to Bardet-Biedl syndrome (Table 3) [15–22]. The encoded C8ORF37 protein is a ciliary protein located at the base of the photoreceptor connecting cilia [16], while its role in modulating retinal function is not fully elucidated. In this study, the patient carrying homozygous nonsense *C8ORF37* mutation presents early onset RP with macular involvement, which is similar to previous reports [15, 17]. *TULP1* mutations are implicated in autosomal recessive RP and LCA etiologies (Table 3) [22–57]. TULP1 protein plays crucial roles in maintaining retinal homeostasis. According to previous reports, TULP1 interacts and co-localizes with F-actin in photoreceptor cells of bovine retina [58], and RPE phagocytosis ability was remarkably reduced in *TULP1*^−/−^ mice [59]. Thus, TULP1 is required for maintaining regular functions of photoreceptors and RPE cells. We herein identified *TULP1* mutations in two siblings demonstrating RP with early onset and quick progression. Further confirmatory functional studies are still needed to better illustrated pathogenesis of the identified novel mutations.

**Table 3:** List of mutations reported in *C8ORF37*, *OFD1* and *TULP1* associated retinopathies

Gene	Variation			Disease	References
	Nucleotide	Amino acid	Domain		
*C8ORF37*	c.155+2T>C	–	–	CRD	[56]
*C8ORF37*	c.156−2A>G	–	–	CRD	[15, 18]
*C8ORF37*	c.243+2T>C	–	–	RP	[21]
*C8ORF37*	c.244−2A>C	–	–	RP	[17]
*C8ORF37*	c.374+2T>C	–	–	EORD	[20]
*C8ORF37*	c.497>A	p.L166*	–	RP	[15, 18]
*C8ORF37*	c.529C>T	p.R177W	–	CRD, BBS	[15, 18, 19, 22]
*C8ORF37*	c.545A>G	p.Q182R	–	RP	[15, 18]
*C8ORF37*	c.555G>A	p.W185*	–	RP	[17], this study
*C8ORF37*	c.575delC	p.T192Mfs*28	–	EORD	[20]
*OFD1*		p.T120A	–	RP	This study
*OFD1*	IVS9+706A>G	p.N313fs*330	Coiled coil domain	RP	[13]
*TULP1*	c.3G>A	p.M1I	–	RP	[25]
*TULP1*	c.99+1G>A	–	–	LCA, RP	[23, 26]
*TULP1*	c.280G>T	p.D94Y	–	LCA	[27]
*TULP1*	c.286_287delGA	p.E96Gfs*77	–	RP	[57]
*TULP1*	c.350−2delAGA	–	–	RP	[28]
*TULP1*	c.394_417del	p.E120_D127del	–	RP	[29]
*TULP1*	c.539G>A	p.R180H	–	LCA	[30]
*TULP1*	c.627delC	p.S210Qfs*27	–	LCA	[31]
*TULP1*	c.629C>G	p.S210*	–	RP	[32]
*TULP1*	c.718+2T>C	–	–	LCA, RP	[33]
*TULP1*	c.725_728delCCAA	p.P242Qfs*16	–	LCA	[34]
*TULP1*	c.901C>T	p.Q301*	Tubby domain	LCA, CRD	[35, 36]
*TULP1*	c.937delC	p.Q301fs*9	Tubby domain	RP	[28]
*TULP1*	c.932G>A	p.R311Q	Tubby domain	RP	[37]
*TULP1*	c.956G>A	p.G319D	Tubby domain	RP	[38]
*TULP1*	c.961T>G	p.Y321D	Tubby domain	LCA	[34]
*TULP1*	c.999+5G>C	–	Tubby domain	LCA, RP	[33]
*TULP1*	c.1025G>A	p.R342Q	Tubby domain	RP	[37]
*TULP1*	c.1047T>G	p.N349K	Tubby domain	RP	[39]
*TULP1*	c.1064A>T	p.D355V	Tubby domain	LCA	[34]
*TULP1*	c.1087G>A	p.G363R	Tubby domain	CRD	[40]
*TULP1*	c.1081C>T	p.R361*	Tubby domain	LCA	[41]
*TULP1*	c.1102G>T	p.G368W	Tubby domain	LCA	[26]
*TULP1*	c.1112+2T>C	–	Tubby domain	RP	[42]
*TULP1*	c.1113–2A>C	–	Tubby domain	LCA	[34]
*TULP1*	c.1138A>G	p.T380A	Tubby domain	LCA, RP	[43, 45, 46]
*TULP1*	c.1145T>C	p.F382S	Tubby domain	RP	[47]
*TULP1*	c.1198C>T	p.R400W	Tubby domain	LCA, RP, CRD	[26, 48, 49]
*TULP1*	c.1199G>A	p.A400Q	Tubby domain	RP	[50]
*TULP1*	c.1204G>T	p.E402*	Tubby domain	LCA	[26]
*TULP1*	c.1224+4A>G	–	Tubby domain	RP	[29]
*TULP1*	c.1246C > T	p.R416C	Tubby domain	RP	[25]
*TULP1*	c.1255C>T	p.R419W	Tubby domain	RP	This study
*TULP1*	c.1258C>A	p.R420S	Tubby domain	RCD	[51]
*TULP1*	c.1259G>C	p.R420P	Tubby domain	RP	[23]
*TULP1*	c.1318C>T	p.R440*	Tubby domain	LCA	[31]
*TULP1*	c.1349G>A	p.W450*	Tubby domain	LCA	[27]
*TULP1*	c.1376T>A	p.I459K	Tubby domain	RP	[23, 24]
*TULP1*	c.1376T>C	p.I459T	Tubby domain	RP	[42]
*TULP1*	c.1376_1377delTA	p.I459Rfs*12	Tubby domain	LCA	[34]
*TULP1*	c.1381C>G	p.L461V	Tubby domain	LCA, RP	[33]
*TULP1*	c.1444C > T	p.R482W	Tubby domain	RP	[44, 48]
*TULP1*	c.1445G>A	p.A482Q	Tubby domain	RP	[46]
*TULP1*	c.1466A>G	p.K489R	Tubby domain	RP	[29, 43, 52, 57]
*TULP1*	c.1472T>C	p.F491L	Tubby domain	RP	[23]
*TULP1*	c.1495+1G>A	–	Tubby domain	RP	[24]
*TULP1*	c.1495+2_1495+3insT	–	Tubby domain	RP	[53]
*TULP1*	c.1495+4A>C	–	Tubby domain	RP	[57]
*TULP1*	c.1496−6C>A	–	Tubby domain	RP	[23, 29]
*TULP1*	c.1511_1521del	p.L504fs*140	Tubby domain	RP	[44]
*TULP1*	c.1518C>A	p.F506L	Tubby domain	LCA	[31]
*TULP1*	c.1561C>T	p.P521S	Tubby domain	RP	[57]
*TULP1*	c.1582_1587dup	p.F528_A529dup	Tubby domain	LCA, RP	[54]
*TULP1*	c.1604T>C	p.F535S	Tubby domain	LCA	[55]

*CRD* cone-rod dystrophy, *RP* retinitis pigmentosa, *EORD* early-onset retinal dystrophy, *BBS* Bardet–Biedl syndrome, *LCA* Leber congenital amaurosis

## Conclusions

In summary, we demonstrate the coexistence of diverse inheritance modes and mutations affecting distinct disease causing genes in two RP families. Our findings reinforce the genetic complexity of RP, provide novel insights into the assessments of complicated pedigrees with consanguinity, and highlight the need for extensive molecular evaluations in such challenging families involving diverse inheritance modes and mutations.

## Additional files

**Additional file 1: Table S1.** Post-filtration variants in family A.

**Additional file 2: Table S2.** Post-filtration variants in family B.

**Additional file 3: Table S3.** Overview of data production.

**Additional file 4: Table S4.** Coverage for all exons in all known RP genes.

## Abbreviations

RP: retinitis pigmentosa
NGS: next-generation sequencing
WES: whole exome sequencing
bp: base pair
LM-PCR: ligation-mediated polymerase chain reaction
RPE: retinal pigment epithelium
